# Exploring Social Interactions in the Context of Justice System Involvement: Perspectives of Patients and Psychiatric Nurses

**DOI:** 10.1177/10547738241253882

**Published:** 2024-05-20

**Authors:** Etienne Paradis-Gagné, Myriam Cader, Dave Holmes, Emmanuelle Bernheim, Janie Filion

**Affiliations:** 1 Université de Montréal, QC, Canada; 2 University of Ottawa, ON, Canada

**Keywords:** justice system involvement, qualitative research, judiciarization, social interactions, mental health

## Abstract

Psychiatric nurses who work with people who are involved with the justice system experience ethical and moral tension arising from their dual role (care and control). This is known to significantly affect the development of a therapeutic relationship between nurses and patients. (a) better understand how justice system involvement affects people living with mental disorders and the nurses who work with them; (b) explore the influence of judiciarization on social interactions between these actors. Grounded theory (GT) was used as the qualitative methodology for this research. Semi-structured interviews were conducted with participants. The study was carried out in three different units of a psychiatric institution: Psychiatric Intensive Care Unit, Emergency Department, and Brief Intervention Unit. A sample of 10 patients and 9 psychiatric nurses was recruited (*n* = 19). Theoretical sampling was used to recruit participants. We followed the iterative steps of qualitative GT analysis (open coding, axial coding, constant comparison, and modelization). Three main themes emerged from the qualitative analysis: (a) Experience of Justice System Involvement, (b) Crisis, (c) Relational Aspects and Importance of the Approach. These results will inform nurses and healthcare providers about the impacts of justice system involvement on people living with mental illness and how clinical practices can be better adapted to this population with complex health needs.

## Introduction

In many countries, it is not uncommon for people with mental health problems to come into contact with the justice system (Bonfine et al., 2020; Hiday & Ray, 2017). Involvement with the justice system can involve legal coercion measures such as involuntary hospitalization [or confinement] (Gouvernement du Québec, 2020), treatment orders, recourse to a specialized mental health court (MHC), and, in the forensic context, a verdict of not criminally responsible after an individual has committed an offense (Antonsson et al., 2023; Government of Canada, 2015; Hiday & Ray, 2017). Numerous issues are associated with the over-representation of people living with mental health problems in the justice system. Studies show that there is a strong stigma attached to people with justice system involvement (Chaimowitz, 2012; Frappier et al., 2009). Racialized and minority groups are particularly affected by judiciarization (National Institute for Health and Care Excellence, 2017; Rotter & Compton, 2022; Sugie & Turney, 2017). For example, in Canada, Indigenous and racialized people with mental health problems are over-represented within the criminal justice system (Chaimowitz, 2012; Watts & Weinrath, 2017). Moreover, the “judiciarization of the mentally ill” (Van Hout et al., 2023) is carried out from a public safety perspective with a broader objective of social control (Konrad & Lau, 2010; Peternelj-Taylor, 2008).

As McKenna et al. (2011) point out, nurses who work with patients under judicial mandate have a central role: “Mental health nurses have always undertaken key positions in services, which interface with legal coercion” (p. 551). While grounded in a philosophy of care and recovery, nurses practicing with individuals subject to judicial measures are also performing the role of social control and coercion (Jager & Perron, 2018), and this dual role of providing care while simultaneously exercising control and discipline can generate ethical and moral tension (Austin et al., 2009; Hellzén et al., 2023; Hörberg & Dahlberg, 2015). The impact on the development of a therapeutic relationship between nurses and patients is well known (McKenna et al., 2011).

The literature shows the difficulty faced by nurses and healthcare professionals who seek to combine the roles of care providers and control agents and how this complicates the establishment of a therapeutic relationship with judiciarized patients (Askola et al., 2017; Dutta et al., 2016; Gildberg et al., 2012; Wittouck & Beken, 2019). Muir et al. (2023) point out the different issues, both clinical and ethical, that psychiatric nurses face when working with legally involved patients. Studies also reveal that patients may perceive they are being coerced, which can affect their desire to receive care and treatment (Akther et al., 2019; Hughes et al., 2009; Tomlin et al., 2020). A number of authors highlight a significant lack of research on interactions and relationships between nurses and patients in psychiatric and forensic contexts (Dutta et al., 2016; Hörberg & Dahlberg, 2015; Marshall & Adams, 2018). This finding is also mentioned by Askola et al. (2018), who describe the paucity of literature on the experience of patients subject to legal measures. More research is needed, however, to ensure that care and services are adapted to the complex needs of these people at a time when mental health resources are increasingly scarce (Snyder et al., 2022). To further explore the issues inherent in this understudied phenomenon, we conducted qualitative research with the aims of (a) better understanding how justice system involvement affects people living with mental disorders and the nurses who work with them, (b) exploring how this context influences the social interactions between these actors. Data from the perspective of patients was the subject of a previous publication (Paradis-Gagné et al., 2023). This article focuses on data collected from the perspective of patients and nurses and examines the overall results of this qualitative research.

## Methodology

***Design*:** Grounded theory (GT), developed by Corbin and Strauss (2014), was used as the qualitative approach for this research. This methodology makes it possible to discern social processes in the collected data (Corbin & Strauss, 2014). Social processes are observed from the accounts of participants who have experienced the phenomenon under study. Theoretically, GT is based on symbolic interactionism, a theory of sociology that emphasizes the importance of social interactions within a given environment (Eaves, 2001). In the context of our study, this methodology was particularly appropriate to our research objectives.

### Sampling

This research was carried out in three different units of a psychiatric institute in Eastern Canada (Psychiatric Intensive Care Unit [PICU], Emergency Department, and Brief Intervention Unit). The theoretical sampling method (Draucker et al., 2007) was used, a method specific to GT that consists of selecting participants from different groups to validate the categories and concepts emerging from the analysis. We recruited a sample of 19 participants (10 patients and 9 nurses) (Tables 1 and 2). We stopped recruiting participants after saturation had been reached, that is, when the emerging categories had been sufficiently developed, and there was no need for more data (O’Reilly & Parker, 2013).

**Table 1.** Patients (*n* = 10).

Characteristics	*N*
Unit	
Emergency Department	7
Brief Intervention Unit	1
PICU	2
Gender	
Male	6
Female	4
Ethnicity	
Black	3
White	5
Hispanic	1
Middle Eastern and North African	1
Experience with the justice system	
Involuntary confinement	6
Treatment order	1
Not criminally responsible	4
Detention (prison)	3

*Note.* PICU = Psychiatric Intensive Care Unit.

**Table 2.** Nurses (*n* = 9).

Characteristics	*N*
Unit	
Emergency Department	3
Brief Intervention Unit	2
PICU	4
Gender	
Female	6
Male	3
Ethnicity	
Black	2
White	6
Middle Eastern and North African	1

*Note.* PICU = Psychiatric Intensive Care Unit.

### Data Collection

Semi-structured individual interviews were conducted with participants. This type of interview allows for flexibility with interview questions, enabling in-depth exploration of the participant’s experience of the phenomenon under study (Corbin & Strauss, 2014). Interview questions focused on three themes: (a) contact with the justice system, (b) perceptions and experience, and (c) relationships and interactions with patients and nurses. The interviews lasted between 20 and 90 min and were conducted in confidential offices in the care unit.

### Data Analysis

The interviews were analyzed qualitatively according to the steps Corbin and Strauss (2014) have suggested for GT (Table 3).

**Table 3.** Qualitative Analysis.

Open coding	Identifying codes in interview transcripts. This step requires a thorough reading of the transcripts (microanalysis) to be able to reduce the data to codes (words, symbols, quotations, or short phrases that synthesize what the participants have said).
Axial coding	Grouping various codes into more general concepts (abstraction) and defining their properties and dimensions. At this stage, it is important to analyze emerging concepts according to their specific contexts (e.g., gender, culture, ethnicity, or social and legal contexts). The result of this step is the identification of central categories through codes.
Constant comparison	Comparison of categories and cases with each other; comparison of emerging results with relevant theoretical literature and conceptual frameworks.
Modelization (or theorization)	Developing a model from core categories. Modelization in GT means (a) identifying the meaning or significance of an event, phenomenon, or situation; (b) linking various elements of a phenomenon in an explanatory schema; (c) renewing our understanding of the phenomenon by shedding new light on it.

*Note.* GT = grounded theory.

### Ethical Considerations

This research was conducted in compliance with ethical standards, and a certificate of approval was obtained from the hospital where the study was conducted (Certificate #FWA00001935 and #IRB00002087). With regard to ethical issues, participants were asked to read and sign a consent form. For patient recruitment only, a gift card was given as compensation. Since the research was carried out with people in vulnerable situations, it was specified in the consent form that the decision not to participate or to withdraw from this study would have no consequences on the quality of care or on the relationship with the teams providing it. Data confidentiality has been respected, and participants’ names have been anonymized.

### Quality Criteria

The criteria for assessing GT quality (Corbin & Strauss, 2014) were followed: (a) methodological consistency: this criterion concerns quality control of the methodology. For this study, we followed all the steps of the GT method and used the appropriate procedures (e.g., theoretical sampling, analysis steps, and theoretical saturation). (b) Credibility: a logbook, analytical memos, and conceptual maps were drawn up to ensure the empirical grounding of the results. We also presented the theoretical framework of this research and the influence of the theories mobilized during data analysis. (c) Sensitivity: throughout the research, we demonstrated sensitivity both to the study participants and to the data collected. In addition, the researchers, through their experience in research in psychiatric care settings, possess advanced knowledge of the methodological and theoretical approaches used. (d) Applicability: this criterion concerns the use of results both in clinical settings (changes in clinical and organizational practices, clinicians’ awareness of the influence of the phenomenon) and in health policies.

## Theoretical Framework

Although GT does not explicitly call for the use of theoretical frameworks in interpreting data, a certain theoretical perspective is integral to the analysis process (Corbin & Strauss, 2014). In this research, we drew on the works of Goffman (1963) and Foucault (2003). Their critical perspectives were particularly useful in elucidating the social dimensions (interactions, relationships, and processes) of the data. According to Goffman, interactions between staff and patients within psychiatric institutions are structured around specific norms. Power and control are also important characteristics of these environments. Foucault (2003) discusses the concept of “psychiatric-judicial mesh” (p. 276). This refers to a set of rules, standards, laws, and clinical practices established from a perspective of care and social control. This *mesh* (or *interface*) enables greater interaction between two domains of power and knowledge: justice and mental health. Drawing on the complementary perspectives of Foucault and Goffman (Hacking, 2004), we made a constant comparison of the results at the analysis stage.

## Results

**Figure 1.**
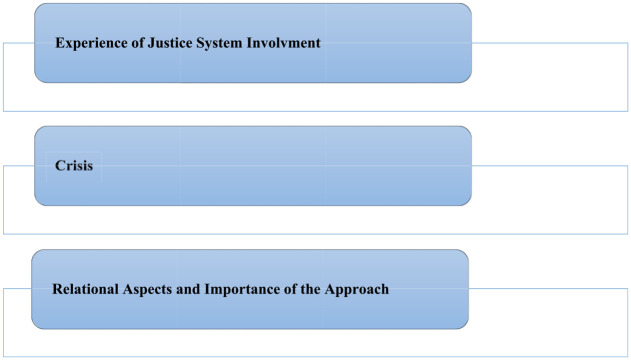
Main themes.

Three main themes emerged from the joint analysis of interviews with nurses (N) and patients (P): (a) Experience of Justice System Involvement; (b) Crisis; (c) Relational Aspects and Importance of the Approach (Figure 1).

### Theme 1: Experience of Justice System Involvement

The first theme to emerge from our data analysis concerns the experience of judiciarization as reported both by individuals subjected to judicial measures and by nurses.

#### Diversity of Experiences

First, we found that people living with mental illness who come into contact with the justice system may be imposed with different measures. Among our sample of participants, non-criminal or civil confinement mechanisms and treatment orders were the most common measures.

When we talk about the justice system, yes, there are those who come in and are under treatment orders, there are those who come in and are in confinement . . . So, yes, we work with this clientele on a daily basis. (N5)

Another situation that nurses regularly encounter is that of patients admitted under a Review Board mandate: these persons find themselves under the jurisdiction of the court after having been declared not criminally responsible on account of mental disorder.

When it comes to the Review Board, it’s because they’ve already committed something criminally, so it could be assault, it could be . . . he’s killed someone—it’s already happened. Otherwise, they’re on confinement because they’re very disorganized, they’re suicidal, they’re self-mutilating, they’re dangerous to themselves or to others. These are cases we often come across. (N9)

Three participants had spent time in prison before being admitted to the psychiatric care unit, and another participant had been referred to a special MHC after assaulting police officers. The justice system experiences of those we interviewed are, therefore, diverse and sometimes overlapping.

I’ve been in prison, I’ve been in hospital. My early life was prison and hospital. Since I left school . . . because I was selling drugs, too. (P7)

One of the findings concerns the high proportion of patients admitted under judicial measures, as indicated by participants in the following interview excerpts. This is, therefore, a central component of nurses’ clinical practice. Nurses working with this clientele must be knowledgeable about the full range of relevant legal procedures, and their conditions and terms of application.

Right now, I think I have more patients in confinement than in free treatment . . . and patients on Review Board. Those on court order, sometimes, for treatment or residential placement. (N1)

I would say that around a third of patients are voluntary. Two thirds are not voluntary, either because they come under the Review Board mandate, or what we call delegations of authority, or because of a treatment order when they already had an order. So they’ve stopped their treatment. Or they may come under confinement. (N3)

#### Challenges and Difficulties

Accounts given by participants subject to legal measures reveal several difficulties encountered by both nurses and patients. For patients, contact with the justice system can be a challenging event due to the coercive nature of judicial measures. Participants also referred to the negative experience of long waiting times. Lengthy legal procedures were another area of concern. A short-term concern mentioned by participants is the duration of confinement, which can sometimes extend to over 20 to 30 days. The length of a treatment order was a major issue in the medium- and long term.

Now it’s three years. They can give you three years. Three years is a long time for a person. Then, they decide on your accommodations, your life and it’s a little more restrictive than confinement. (N2)

The same applies to people admitted under a Review Board mandate, who often find themselves waiting for a place in a forensic unit, which considerably lengthens waiting times.

These patients [in confinement] are kept for an average of 5 to 10 days, depending on the case. Then there’s where patients are admitted by the psychiatrist because, first of all, they may be patients on Review Board who require longer-term hospitalization. (N6)

In the [intensive] unit here I’ve been here for 3 months because they’re waiting for a transfer to the [forensic hospital] or in therapy. [. . .] But it’s still a long time. It’s tough, I find it tough. (P10)

You just want to pull the hair out of your head, it’s just long, it’s very long. . . if maybe there were activities you could do. Like I love doing my painting, I love playing music instruments. But we’re not allowed to do anything. I’m not even allowed to use the WIFI. (P1)

#### Comprehension of the Legal Process

Another reality that emerges from the data concerns comprehension of the legal process. Lack of access to information and incomprehension are major irritants for patients.

To get answers to your questions and have things explained, well, there’s no one here at this time to offer you that kind of help, to be able to explain the terms, what it means, where we’re going, and how it’s done. That made it difficult too. (P1)

As part of their role, nurses must provide patients with ongoing information on their rights and the steps involved in the legal process (e.g., court appearances and the possibility of contesting legal measures). This is complicated, however, when an individual’s mental state is impaired.

### Emotional Responses

Participants mentioned experiencing various emotions when confronted with the judicial process in general and the coercive measures imposed in psychiatric settings.

Of course, when a patient doesn’t want to stay, it’s emotional management . . . he goes to the judge. The judge validates confinement. So there’s an obligation to stay in hospital. This creates difficult moments for the patient. (N1)

The deprivation of freedom in the psychiatric unit is a significant problem. For many participants, finding themselves hospitalized against their will can feel as though they were in prison.

We see that a patient who is in confinement, he or she sees that there’s a loss of control, a loss of power that he or she feels. And they’re frustrated. You can see that they’re not happy. They’re irritable about the fact that they’re being held against their will. Unfortunately. (N3)

The data also reveal participants’ experience of anxiety and stress, for example, stress due to incomprehension and being faced with the unknown, particularly when it is their first contact with the judicial or psychiatric system.

There’s the whole context around it, especially if it’s a first visit, the shock of being brought to hospital against their will. It brings upheavals. (N6)

Patients are usually stressed because they’re under confinement. They come here in a state of stress, especially when there is an order for a psychiatric examination. And sometimes we have patients who don’t understand what’s happened either. (N2)

The anxiety associated with the stigma of this judiciarization (particularly in a forensic context) is also a heavy burden. They are now stigmatized for being both mentally ill and criminalized.

I feel like I have a sticker on my forehead that says: “He’s just been released from the forensic hospital.” (P10)

### Theme 2: Crisis

This second theme refers to the crisis associated with patients’ contact with the justice system. Aggravation of symptoms combined with the risk of violence toward oneself or others means they must be held against their will. Substance use, when combined with aggravation of their mental condition, can also accentuate the crisis and state of psychosis.

Sometimes when I smoked, things got out of control. (P7)

As is the case with substance use, the perception of mental illness influences the judicial process. The use of coercive measures (involuntary admission and treatment) often results from patients’ lack of acknowledgment of their illness and deteriorating mental health. The denial of symptoms can play an important role both in nurses’ practice and the experience of patients who are subject to legal measures.

When a patient knows that there’s a request for confinement, or when he goes to see the judge and then comes back and there’s a judgment, it’s certainly rarely positive. You have to deal with the patients’ reactions, and sometimes their incomprehension, because it’s often: “I’m not sick, I don’t need to be here!” So that’s a big part of our job. (N8)

[The court] will give priority to my psychiatrist’s version. My psychiatrist tells me that supposedly I’m a bipolar person and for this reason I have to continue like this and extend the follow-up with her for another year. (P8)

Even though many patients do not acknowledge the existence of mental illness, those who are familiar with the mental health and justice system “play the game,” accepting judicial measures resulting in hospitalization or involuntary treatment. Unlike those in their first episode, those who have been in the psychiatric-judicial system for several years are much more acquainted with the court.

When it’s the first time, they don’t understand. They say, “But why? I didn’t do anything. I haven’t done anything. Why are you taking me to court?” And they think it’s for criminal cases and things like that. We try to explain it to them. But they don’t always understand that it’s a question of their right to freedom, their fundamental rights . . . But the more often a person is hospitalized, the more often they have to deal with psychiatric confinement. At that point, he knows the gimmick. He knows the game. He knows the system. (N3)

It was my first time [in confinement], I didn’t know how to proceed if there were any questions to answer. I had no idea. I had no idea. (P1)

As we can see, those whose involvement with the legal system continues over longer periods of time develop a degree of familiarity with ways of doing things and procedures, which exacerbates the risk of the person being institutionalized or, as Nurse 2 points out, “chronicized.”

Sometimes, for people on treatment orders and those on Review Board, I find it a little more difficult. Because these are people who are somewhat chronicized in the system. (N2)

This involves a transition from an initial crisis state to a certain chronicity for the person over the long term.

### Theme 3: Relational Aspects and Importance of the Approach

In this third theme, the data show that relational aspects between nurses and patients in the context of legal proceedings can be complex.

#### Relational Issues

The development of a relationship of trust is different when people find themselves in a situation of involuntary care as opposed to when they are seeking care.

When they come with these elements [legal status], it’s even harder for the alliance to work with these people. To enable them to work with us. To take treatments, to work together to shorten hospitalization. (N3)

To establish such an alliance, it is first important to develop a level of trust. When trust is gradually established, it becomes possible to work with the person on his or her recovery.

I’m more comfortable when I trust the person. Period. Like, if I don’t trust the person, it’s hard for me to get treatment. (P4)

I’m fine with the caregivers, some I’m more attached to than others. [. . .] I try to be close to the doctors, I create bonds with them, otherwise it would be long and boring. I do have a nice bond with certain people. (P10)

A concept that emerged from the analysis is that of “decisional balance.” Some of the nurses we interviewed were torn between two roles: that of helping and being there for the person and that of applying restrictive measures when the patient is involuntarily admitted or treated.

Generally, last resort is not what we want. We take all the time we need to explain. But when a patient can’t listen, what can you do? You can’t let him go, and you can’t let him hit anyone either. You have to intervene by other means, which are also restrictive and not always appreciated by patients. (N5)

The decisional balance raises ethical questions for nurses, who sometimes find themselves assuming two different roles (caregiving vs. control/security role). To mitigate the deleterious effects on the development of a bond of trust, some nurses adopt the strategy of taking a neutral and impartial posture when they must apply judicial measures.

Like it or not, we’re kind of in the role of: “We’re just here to take care of you. So if you’re on confinement, it’s not on us. We didn’t decide to put you in confinement, the judge did.” We can always say that . . . So, in a way, we’re playing with that to our advantage. (N3)

#### Approach to Patient Care

The approach nurses adopt with patients is central to developing a bond of trust. Considering the person, being honest and transparent, and remaining empathetic are some of the important elements in establishing an alliance mentioned by the nurses we interviewed.

The approach is very important. The patient who’s on confinement, if you can start by understanding his problem. In other words, “I understand you, being here is not pleasant, it’s not pleasant for anyone. Being here against your will, it can’t be fun for you. I couldn’t agree more.” (N5)

It’s the staff’s approaches that make all the difference. Because you don’t want to be told just anything when you’re already in an unfamiliar situation. People don’t understand that sometimes it’s better to be direct and say, “No, ma’am, we don’t know, we don’t know, I can’t help you.” (P1)

With regard to interventions, nurses spoke of the importance of listening to people’s distress and frustration.

What I can offer them as a care provider is a listening ear. In other words, I can hear the violence they feel at finding themselves here without their consent. In fact, as a human being, I can hear that it’s very difficult to be stuck in a hospital. (N1)

The nurse who cares for me now is wonderful. She always listens, she does a lot of talking with her patients, she talks, and if there’s something we don’t understand, she’s not going to make us look uneducated or stupid or anything. She’ll take her time, and that makes all the difference in the world, I think. (P1)

Last, according to the participants we met, the ability to establish a relationship of trust with people being treated against their will also depends on clinical experience. To work with this population, nurses need to be familiar with mental illness and the justice system, whether in a civil or forensic psychiatric context.

It takes knowledge. You have to know the procedures. Of course, when nurses arrive here, they don’t know the definitions of each of the legal status. If we’re talking about the confinement, for example. What’s a temporary confinement, versus a preventive one? When do you have to hurry up and make a report? [. . .] It requires a good understanding. (N3)

Experience is also very important. Often, it’s just based on what you’ve already seen. The way you can resolve a crisis with a patient. And that’s something you learn on the field. [. . .] Experience is acquired over time. Secondly, the sharing of knowledge between peers is also very important. (N5)

This clinical experience can be acquired over time through patient contact and peer mentoring.

## Discussion

We drew on GT to study how inpatients who had come into contact with the justice system interact with nurses and how nurses intervene with them from a therapeutical perspective. We have seen that the judicial trajectories of people admitted in psychiatric settings can be diverse, and they have an impact on nursing practice. Moreover, participants may experience these trajectories differently. Also, our results indicate that a significant proportion of patients are subject to judicial measures, particularly involuntary confinement, treatment orders, and Review Board mandates (i.e., verdict of non-responsible on account of mental disorder). For nurses, this is no longer an anecdotal and exceptional practice but rather the daily routine. In this respect, the literature shows that psychiatric nurses, in their pivotal role as members of mental healthcare teams, are increasingly central players in the implementation of judicial measures (Galon & Wineman, 2010; Laing, 2012). For Muir et al. (2023), a “greater reliance on nurses to deliver key functions under mental health law” is emerging internationally (p. 815). On a conceptual level, Michel Foucault, who studied issues of psychiatry and social control, states that psychiatry and justice are apparatuses (*dispositifs*) of power that intersect and increasingly influence each other throughout our societies. “At this point psychiatry becomes medico-judicial not just at its limits and in exceptional cases, but all the time, in its daily life and working agenda” (Foucault, 2003, p. 163).

A central finding of this study is the complexity of social interactions between nurses and patients in the context of justice system involvement. This is in keeping with Wittouck and Beken (2019), who, in a critical review of the literature, reported on how this situation can significantly affect the development of a therapeutic alliance with healthcare professionals. The finding is present across the literature (Donnelly et al., 2011; Muir et al., 2023). It is nonetheless possible to develop a bond, a therapeutic alliance, with patients who are subject to restrictive measures, as reported by the participants in our study.

In institutional settings, the relationship between patients and care providers can be significantly affected by the coercive legal framework in place. In this context, nursing care is provided with the aim of recovery within a regulatory framework focused on control and public safety. This duality complicates the development of a relationship of trust with patients. Nurses find themselves acting from two seemingly dichotomous poles: coercion/restraint and care/treatment. Other studies on this dual role in psychiatric nursing have reached similar conclusions to ours (Dutta et al., 2016; Hellzén et al., 2023; Jacob, 2012; Marshall & Adams, 2018). The qualitative study by Merkt et al. (2021) points out the presence of a “dual loyalty conflict” experienced by healthcare professionals in the context of court-mandated treatment. According to these authors, the burden of loyalty sets the work of building patients’ trust and the therapeutic relationship against the obligation of upholding legal measures and imposing more coercive measures such as forced hospitalization and treatment. Similarly, the concept of “decisional balance” emerged from our study to describe the delicate balance between (a) caring and doing no harm and (b) ensuring the safety of the person and the environment.

A study conducted among forensic nurses by Gildberg et al. (2012) highlights key aspects of nurses’ interactions with involuntarily admitted patients: active listening, use of humor, respect, honesty, availability, and a non-judgmental approach. While these things may appear obvious, they remain central to building trust and can often be neglected in an environment where a coercive and carceral culture predominates (Jacob & Holmes, 2011). The nurses interviewed by Salzmann-Erikson et al. (2016) also report the importance of informal exchanges and leisure social activities to “getting to know the person through interacting” (p. 1430). As part of a qualitative systematic review of the experience of patients subject to mental health legislation, Akther et al. (2019) identified boredom and a lack of social, recreational, and occupational activities as issues particularly reported in the literature. In terms of social interaction, the patients we interviewed also emphasized lack of activities, boredom on the unit, and the need for more interaction with staff.

Another finding of our study concerns the development of a judiciarized person’s “career” over the years and the risk of their becoming institutionalized. Indeed, being subject to repeated judicial measures can place the person on what sociologist Goffman (1963) defines as the “moral career of the mental patient” (p. 117). Goffman has studied how this moral career is shaped by contact with the psychiatric environment and how it restructures the individual’s social interactions with care staff in institutional settings—particularly in the context of compulsory admission.

The inpatient learns to orient himself in terms of the “ward system.” In public hospitals this usually consists of a series of graded arrangements built around wards, administrative units called services, and parole statuses. [. . .] The institutionalization of these radically different levels of living throws light on the implications for self of social settings. (pp. 137–138)

Like Goffman, Foucault (2003) affirms that judiciarization places people living with mental illness on a career, a permanent state of abnormality for those considered “at risk” and who must be subject to coercive measures. Nursing scholar Perron (2012) also used the concept of the moral career to analyze data from an ethnographic study conducted in a forensic setting. This author highlighted how entry into an institutional environment restructures the identity of admitted patients and how this affects nurse-patient interactions. In this context, psychiatric culture and its institutional rules (closed units, security protocols, searches of personal belongings, application of control measures such as seclusion and restraint, and PRN medication) significantly affect the lived experience of involuntarily admitted patients, as does the crisis they experience. Nurses need to be aware of these realities to avoid perpetuating the retraumatization of those they are caring for. Finally, our findings point to the need for further research to better understand how nurses, healthcare professionals, and service users interact in the context of justice system involvement—considering the increasing application of legal measures such as involuntary admission and treatment. A better understanding of how to provide care, support, and advocate for these vulnerable populations is needed.

## Relevance for Clinical Practice

The implications of this study underline the need for increased professional training: nurses working with people who have come into contact with the justice system need to develop knowledge of the imposed legal measures. The importance of advanced continuing education for nurses working at the interface of mental health and the court system is also raised by Muir et al. (2023). Our results also show that despite patients’ lack of acknowledgment of their illness and need for psychiatric treatment, active listening, consideration for the person, and presence with the patient (through activities and discussions) remain central in nursing practice. Providing patients with information about upcoming legal steps and the judicial process is a key aspect of nurses’ interventions. For example, patients should be informed about the duration of involuntary admission, how the court works, and the possibility of appealing the court order. In this matter, our results are consistent with the clinical recommendations suggested by Galon and Wineman (2010) in the context of a community treatment order and legal coercion.

## Limitations

For this study, we met only inpatient participants, although the issues related to justice system involvement also affect people in outpatient settings and community health nurses (e.g., nurses practicing in assertive community teams). Moreover, this research focuses on the context of the province of Quebec, Canada, which has its own specificities in terms of mental health legislation (Frank et al., 2020). This regulatory framework may diverge from certain other jurisdictions and countries, where the legal measures in place may be quite different.
